# Molecular dissection of RbpA-mediated regulation of fidaxomicin sensitivity in mycobacteria

**DOI:** 10.1016/j.jbc.2022.101752

**Published:** 2022-02-19

**Authors:** Jerome Prusa, Dennis X. Zhu, Aidan J. Flynn, Drake Jensen, Ana Ruiz Manzano, Eric A. Galburt, Christina L. Stallings

**Affiliations:** 1 Department of Molecular Microbiology, Washington University School of Medicine, St. Louis, Missouri, USA; 2 Department of Biochemistry and Molecular Biophysics, Washington University School of Medicine, St. Louis, Missouri, USA

**Keywords:** RNA polymerase, mycobacteria, bacterial transcription, antibiotic action, bacterial genetics, RbpA, fidaxomicin, BL, basic linker, CD, core domain, DMSO, dimethyl sulfoxide, Fdx, fidaxomicin, NTT, N-terminal tail, RNAP, RNA polymerase, RbpA, RNA polymerase binding protein A, RP_o_, RNAP-promoter open complex, SID, sigma interaction domain

## Abstract

RNA polymerase (RNAP) binding protein A (RbpA) is essential for mycobacterial viability and regulates transcription initiation by increasing the stability of the RNAP-promoter open complex (RP_o_). RbpA consists of four domains: an N-terminal tail (NTT), a core domain (CD), a basic linker, and a sigma interaction domain. We have previously shown that truncation of the RbpA NTT and CD increases RP_o_ stabilization by RbpA, implying that these domains inhibit this activity of RbpA. Previously published structural studies showed that the NTT and CD are positioned near multiple RNAP-σ^A^ holoenzyme functional domains and predict that the RbpA NTT contributes specific amino acids to the binding site of the antibiotic fidaxomicin (Fdx), which inhibits the formation of the RP_o_ complex. Furthermore, deletion of the NTT results in decreased *Mycobacterium smegmatis* sensitivity to Fdx, but whether this is caused by a loss in Fdx binding is unknown. We generated a panel of *rbpA* mutants and found that the RbpA NTT residues predicted to directly interact with Fdx are partially responsible for RbpA-dependent Fdx activity *in vitro*, while multiple additional RbpA domains contribute to Fdx activity *in vivo*. Specifically, our results suggest that the RP_o_-stabilizing activity of RbpA decreases Fdx activity *in vivo*. In support of the association between RP_o_ stability and Fdx activity, we find that another factor that promotes RP_o_ stability in bacteria, CarD, also impacts to Fdx sensitivity. Our findings highlight how RbpA and other factors may influence RNAP dynamics to affect Fdx sensitivity.

*Mycobacterium tuberculosis* is the causative agent of the disease tuberculosis, which resulted in an estimated 1.5 million deaths worldwide in 2019 (https://www.who.int/publications/i/item/9789240013131). New strategies are necessary to fight this global health crisis, including the development of novel therapies. Bacterial transcription is a druggable essential process in *M. tuberculosis*, demonstrated by the transcription inhibitor rifampicin’s continued status as a cornerstone of tuberculosis treatment. Bacterial transcription is carried out by an RNA polymerase (RNAP) comprised of five subunits (α_2_ββ’ω), referred to as the core RNAP, and a sixth dissociable subunit (σ) that when bound to core RNAP forms a complex termed the RNAP holoenzyme. Mycobacterial transcription initiation *in vivo* also requires two additional essential RNAP-interacting proteins, RbpA and CarD (1, 2, 3, 4, 5, 6). RbpA and CarD regulate transcription initiation by binding to the RNAP and modulating the kinetics of RNAP-promoter open complex (RP_o_) formation and RNAP promoter escape (2, 5, 6, 7, 8, 9, 10).

RbpA is comprised of four structural domains, including the N-terminal tail (NTT), core domain (CD), basic linker (BL), and sigma interaction domain (SID) (4, 10, 11). Most of the characterization of RbpA has focused on the BL and SID. The RbpA SID domain directly interacts with σ region 1.2, σ nonconserved region, and σ region 2.3 in group I (*M. tuberculosis* σ^A^) and group II (*M. tuberculosis* σ^B^) σ factors (4, 5, 10, 12, 13, 14). The SID domain is both necessary and sufficient for RbpA to associate with the RNAP holoenzyme (5). An arginine at position 88 in the *M. tuberculosis* RbpA SID is critical for the interaction with σ^A^ and σ^B^ (5, 15). The *M. tuberculosis* RbpA BL contains several positively charged residues, including K73, K74, K76, and R79, that are positioned to interact with the negatively charged DNA phosphate backbone near the upstream edge of RP_o_ (4, 10). Alanine substitution at either R79 in the BL or R88 in the SID has demonstrated that the interactions between RbpA and the RNAP and DNA are necessary for RbpA to increase RP_o_ stability during transcription initiation (5, 10, 14). *In vivo*, R79A or R88A substitutions in RbpA result in upregulation of some genes and downregulation of other genes, suggesting that the outcome of RbpA activity may be promoter dependent, possibly due to differences in the kinetics of transcription initiation at each promoter (5, 16, 17).

Much less is known about the functions performed by the RbpA NTT and CD. Deletion of the RbpA NTT increases the ability of RbpA to stabilize RP_o_, and deletion of both the RbpA NTT and CD further increases RP_o_ stability, indicating that both domains antagonize RbpA-mediated stabilization of RP_o_ (5, 10). Structural analysis of RbpA bound to the *M. tuberculosis* RNAP-σ^A^ RP_o_ shows that the RbpA NTT is positioned near the RNA exit channel, possibly contacting the RNAP β switch 3 region (Sw3), β flap, β′ lid, σ^A^ region 3.2 (σ^A^_3.2_, also referred to as the σ “finger” domain), and the β′ zinc binding domain (ZBD), while the RbpA CD is positioned near the RNAP β′ zipper and RNAP β′ ZBD (10, 11). These RNAP structural domains have been characterized to varying levels in *Escherichia coli*, which lacks RbpA. The RNAP β Sw3 is one of five switch regions that are thought to undergo conformational changes during transcription initiation (18). RNAP β Sw3 is positioned near the template DNA −3 and −4 nucleotides, raising the possibility that RNAP β Sw3 could play a role in DNA template strand positioning (19). The RNAP β flap, which includes the flap tip helix that interacts with σ region 4, is important for positioning σ region 4 for interaction with the −35 element of the promoter (20) and represents a common binding interface for transcription factors that directly interact with σ (21, 22). The RNAP β′ lid separates the RNA/DNA hybrid as part of the RNA exit channel and is required for RP_o_ stability and transcription in *E. coli* and *Thermus aquaticus* (23, 24). RNAP σ^70^_3.2_ plays a role in initiating nucleotide triphosphate binding by positioning the DNA template strand for interaction with −4 and −5 nucleotides of the DNA template strand, which affects abortive transcription and promoter escape (25, 26, 27, 28). Both the RNAP β′ ZBD and β′ zipper facilitate RP_o_ formation on promoters with −35 elements that form weak interactions with σ by making promoter contacts within the spacer region between the −10 and −35 motifs (29, 30).

The positioning of the RbpA NTT and CD near multiple different structural and functional domains of the RNAP-σ^A^ holoenzyme implies that the RbpA NTT and CD could impact RNAP activity through a number of mechanisms. However, it is unclear what contacts between the RbpA NTT/CD and the RNAP mediate the antagonism of RP_o_ stability. In addition, structural studies indicate that the RbpA NTT is positioned in the RNAP-σ^A^ holoenzyme complex in such a way that it contributes to the binding site for the antibiotic fidaxomicin (Fdx) (11), which is used to treat *Clostridium difficile* infections. Fdx inhibits transcription initiation by binding the RNAP and blocking the closing of the RNAP clamp that occurs during RP_o_ formation (11, 31). Deletion of the RbpA NTT decreases sensitivity of *M. tuberculosis* RNAP to Fdx *in vitro* and *in vivo* (11), which is proposed to be due to the loss of RbpA’s contribution to the RNAP-Fdx binding interface. However, given that RbpA NTT also decreases RP_o_ stability (5) and is predicted to interact with σ^A^_3.2_, which is known to affect Fdx activity (11, 32), it is possible that RbpA may impact Fdx activity by additional mechanisms. In this study, we interrogate the roles played by residues within the NTT in RbpA-dependent Fdx sensitivity and find that the amino acids predicted by the structural studies to interact with Fdx do partially contribute to Fdx activity *in vitro*. However, we also find that RbpA’s impact on Fdx activity *in vivo* extends beyond the role of the NTT in binding the antibiotic, revealing a dominant contribution for RNAP conformation in Fdx sensitivity.

## Results

### RbpA E17 and R10 synergize to promote Fdx activity against M. tuberculosis RNAP-σ^A^*in vitro*

**Figure 1:**
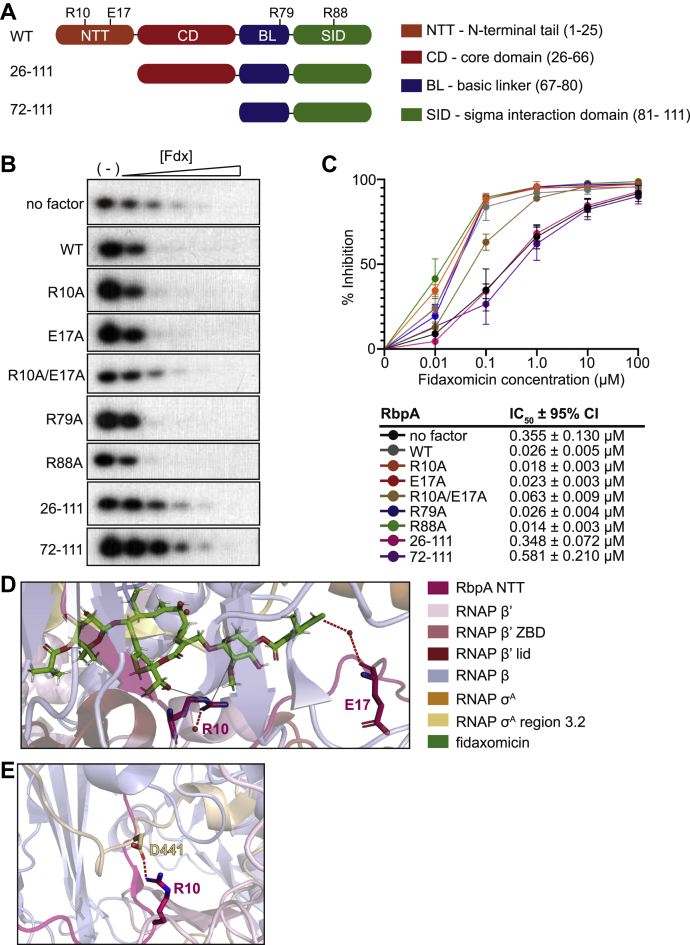
**RbpA E17 and R10 synergize to promote Fdx activity against *M. tuberculosis* RNAP-σ**^**A**^***in vitro*.***A*, schematic of RbpA’s four domain structure including the location of the substituted residues, R10, E17, R79, R88, and the two *M. tuberculosis* truncation mutants, RbpA 26–111 lacking the NTT, and RbpA 72–111 lacking the NTT and CD. *B*, representative gels showing Fdx (0 μM, 0.01 μM, 0.1 μM, 1.0 μM, 10 μM, and 100 μM) inhibition of *M. tuberculosis* RNAP-σ^A^ production of three nucleotide transcripts alone or in complex with RbpA_*Mtb*_^WT^, RbpA_*Mtb*_^R10A^, RbpA_*Mtb*_^E17A^, RbpA_*Mtb*_^R10A/E17A^, RbpA_*Mtb*_^R79A^, RbpA_*Mtb*_^R88A^, RbpA_*Mtb*_^26–111^, or RbpA_*Mtb*_^72–111^ from a linear dsDNA template containing positions −80 to +70 of *M. tuberculosis rrn*AP3 (relative to the +1 transcription start site). *C*, dose–response curves of the experiments shown in (*A*). The curves are generated from at least four replicates from at least two different experiments. Percent inhibition at each Fdx concentration included in the plots compared to no drug is depicted as the mean ± SD. The IC50 for each replicate was calculated by nonlinear regression analysis with four-parameter (EC50, Hill Slope, top and bottom curve plateaus) fitting of log transformed Fdx concentration *versus* normalized response, with the mean IC50 and 95% confidence interval listed in the table. *D*, structural modeling of Fdx binding pocket on the RbpA-bound *M. tuberculosis* RNAP-σ^A^ from PDB structure 6BZO. Fdx and RNAP residues involved in the RNAP-Fdx binding interface are shown with PyMol stick representation while the rest of the structure is shown with PyMol cartoon representation. Polar interactions are indicated by *red dashed lines*, and potential van der Waals interactions are shown as *gray lines*. *E*, structural modeling of RbpA-bound *M. tuberculosis* RNAP-σ^A^ from PDB structure 6C04. RbpA R10 and RNAP σ^A^ D441 are shown with PyMol stick representation while the rest of the structure is shown with PyMol cartoon representation. The polar interaction between RbpA R10 and RNAP σ^A^ D441 is indicated by the *red dashed line*. CD, core domain; Fdx, fidaxomicin; NTT, N-terminal tail; RbpA, RNA polymerase binding protein A; RNAP, RNA polymerase.

*In vitro* assays that monitor the production of a 3-nucleotide product as a proxy of RP_o_ stability have shown that addition of Fdx to *M. tuberculosis* RNAP-σ^A^ holoenzymes reduces the amount of RP_o_ formed following the subsequent addition of NTPs and a DNA template harboring the *M. tuberculosis rrnA*P3 promoter (11). We used this assay with a range of Fdx concentrations to calculate the concentration of Fdx that inhibits 50% of RP_o_ (IC50) formed by RNAP-σ^A^ on the *rrnA*P3 promoter in the presence or absence of different RbpA variants. Addition of WT RbpA_*Mtb*_ (RbpA_*Mtb*_^WT^) to the RNAP-σ^A^ holoenzyme increases the sensitivity of the RNAP-σ^A^ holoenzyme to Fdx in this assay, and this is dependent on the presence of the NTT (deleted in the RbpA_*Mtb*_^26–111^ and RbpA_*Mtb*_^72–111^ mutants) (11) (Fig. 1, *A*–*C*). Deletion of both the RbpA NTT and CD resulted in an IC50 within the confidence interval of the IC50 when the NTT alone was deleted (Fig. 1, *B* and *C*), indicating that the presence of the RbpA CD does not affect Fdx activity against *M. tuberculosis* RNAP-σ^A^ *in vitro*. In contrast, an R88A substitution in the RbpA SID that weakens the interaction between RbpA and the RNAP resulted in an IC50 that was lower and outside the confidence interval, compared to RbpA_*Mtb*_^WT^, suggesting that domains outside of the NTT could increase Fdx sensitivity (Fig. 1*C*). Importantly, a saturating concentration of RbpA protein was used in these assays, and therefore, the different effects of RbpA variants on Fdx sensitivity in this assay should not be a result of altered proportions of RbpA-bound RNAP-σ^A^ complexes.

Structural studies predicted that the NTT contributes contacts with Fdx when the antibiotic is bound to the *M. tuberculosis* RNAP-σ^A^ holoenzyme (PDB: 6BZO), specifically through a water-mediated interaction between RbpA E17 and Fdx (Fig. 1*D*) (11). To determine whether the predicted interaction between Fdx and RbpA E17 underpins NTT-dependent Fdx activity, we calculated the IC50 of Fdx in the presence of RbpA_*Mtb*_^WT^ *versus* an RbpA_*Mtb*_^E17A^ mutant protein. The activity of Fdx against the *M. tuberculosis* RNAP-σ^A^ in the presence of RbpA_*Mtb*_^E17A^ was nearly equal to Fdx activity against the *M. tuberculosis* RNAP-σ^A^ in the presence of RbpA_*Mtb*_^WT^, indicating that alterations in the size and charge of the amino acid side chain at RbpA NTT position 17 do not impact Fdx activity against the *M. tuberculosis* RNAP-σ^A^ (Fig. 1, *B* and *C*).

The structure in Boyaci *et al.* (11) also highlights potential van der Waals interactions between RbpA R10 and Fdx in the RNAP-σ^A^ holoenzyme bound to double stranded forked DNA (PDB: 6BZO) (Fig. 1*D*); however, given the distance between RbpA R10 and Fdx, one would predict this to be a weak interaction. In a separate structure of RbpA bound to *M. tuberculosis* RNAP-σ^A^ in complex with two double-stranded forked DNA molecules that mimics the RP_o_ (PDB: 6C04), the RbpA R10 positively charged side chain is positioned within 2.4 Å of the negatively charged side chain of σ^A^_3.2_ D441, forming a polar interaction (11) (Fig. 1*E*). Fdx activity against *E. coli* RNAP-σ^70^ holoenzyme lacking σ^70^_3.2_ is attenuated approximately 20-fold (32), indicating that σ^70^_3.2_ contributes to Fdx inhibition of the *E. coli* RNAP. Therefore, if RbpA R10 interacts with σ^A^_3.2_, this may also affect Fdx activity. To examine whether RbpA R10 contributes to *M. tuberculosis* RNAP-σ^A^ Fdx sensitivity, we measured Fdx IC50 against the *M. tuberculosis* RNAP-σ^A^ in the presence of RbpA_*Mtb*_^R10A^. Similar to the RbpA_*Mtb*_^E17A^ mutant, we observed no change in Fdx IC50s against the *M. tuberculosis* RNAP-σ^A^ in the presence of RbpA_*Mtb*_^R10A^ compared to RbpA_*Mtb*_^WT^ (Fig. 1, *B* and *C*), indicating that the R10 residue is not required for RbpA NTT-dependent Fdx activity. To determine the effect of disrupting the contacts made by the both RbpA E17 and R10, we measured the Fdx IC50 against *M. tuberculosis* RNAP-σ^A^ in the presence of RbpA_*Mtb*_^R10A/E17A^. Mutating both the R10 and E17 residues resulted in an approximately 3-fold increase in the Fdx IC50 compared to RbpA_*Mtb*_^WT^, although this was still at least 5-fold lower than RbpA mutants lacking the entire NTT (RbpA_*Mtb*_^26–111^ and RbpA_*Mtb*_^72–111^) (Fig. 1, *B* and *C*). These data indicate that loss of one of these residues increases the importance of the other for Fdx activity, but additional mechanisms also contribute to NTT-dependent Fdx activity *in vitro*.

### Multiple RbpA domains impact Fdx activity in vivo

**Figure 2:**
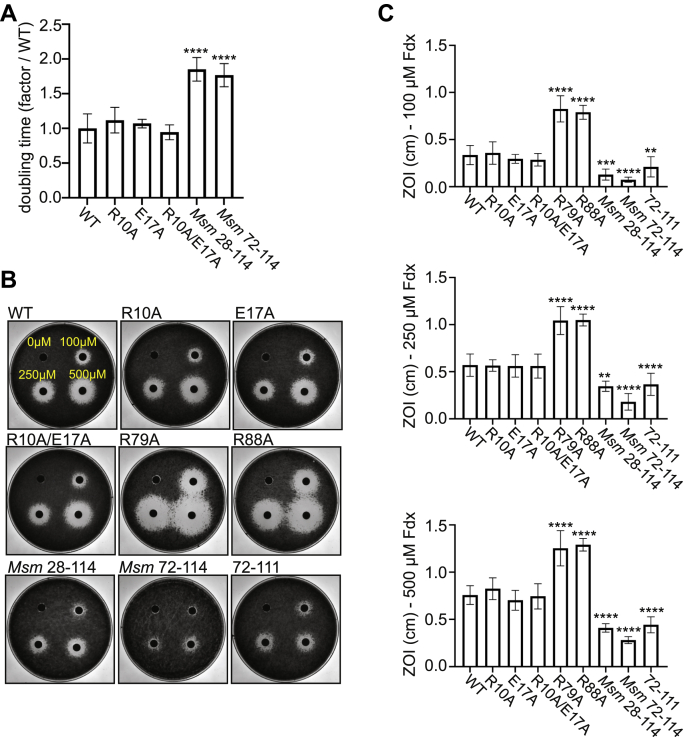
**Multiple RbpA domains impact Fdx activity *in vivo*.***A*, ratio of the doubling times of *M. smegmatis* strains expressing RbpA_*Mtb*_^R10A^, RbpA_*Mtb*_^E17A^, RbpA_*Mtb*_^R10A/E17A^, RbpA_*Msm*_^28–114^, or RbpA_*Msm*_^72–114^ as compared to the average doubling time for the strain expressing RbpA_*Mtb*_^WT^. The mean ± SD from at least two independent experiments with three replicates per experiment. *B*, zones of inhibition (ZOI) by Fdx on bacterial lawns of *M. smegmatis* expressing RbpA_*Mtb*_^WT^, RbpA_*Mtb*_^R10A^, RbpA_*Mtb*_^E17A^, RbpA_*Mtb*_^R10A/E17A^, RbpA_*Mtb*_^R79A^, RbpA_*Mtb*_^R88A^, RbpA_*Msm*_^28–114^, RbpA_*Msm*_^72–114^, or RbpA_*Mtb*_^72–111^ as the only copy of *rbpA*. *C*, mean radii of ZOI ± SD from at least two experiments with at least three replicates at 100 μM, 250 μM, and 500 μM Fdx is plotted. For *A* and *C*, statistical significance of differences was analyzed by ANOVA and Tukey’s multiple comparison test. ∗∗*p* < 0.01; ∗∗∗*p* < 0.001; ∗∗∗∗*p* < 0.0001. All comparisons to RbpA_*Mtb*_^WT^ were included in the analysis, but only statistically significant comparisons are indicated in the figure. Fdx, fidaxomicin; RbpA, RNA polymerase binding protein A; RNAP, RNA polymerase.

Previous work showed that truncation of the RbpA NTT decreases the sensitivity of *Mycobacterium smegmatis* to Fdx (11). To investigate the effect of mutations in RbpA on Fdx sensitivity *in vivo*, we used a strain we previously engineered that expresses *rbpA*_*Mtb*_^WT^ at the *attB* site of *M. smegmatis* and has the endogenous *rbpA* gene deleted (5). We then attempted to replace the *rbpA*_*Mtb*_^WT^ gene at the *attB* site in *M. smegmatis* with alleles encoding each of the RbpA mutants studied in Figure 1 using a gene swapping method (5, 33, 34). We have previously used this approach to generate an *M. smegmatis* strain expressing *rbpA*_*Mtb*_^72–111^, which has a deletion of both the NTT and CD (Fig. 1*A*), as its only *rbpA* allele (5). However, we were unable to generate a viable strain expressing *rbpA*_*Mtb*_^26–111^, which deletes only the NTT (Fig. 1*A*), in place of *rbpA*_*Mtb*_^WT^. In contrast, we were able to replace the *rbpA*_*Mtb*_^WT^ allele with the *M. smegmatis* allele *rbpA*_*Msm*_^28–114^, which has previously been used to study the NTT in *M. smegmatis* (10, 11). Similar to our previous report with the *M. smegmatis* strain expressing RbpA_*Mtb*_^72–111^ (5), RbpA_*Msm*_^28–114^ and RbpA_*Msm*_^72–114^ strains also exhibited a slow growth phenotype (Fig. 2*A*), confirming that while the NTT and CD are not required for viability in *M. smegmatis*, they are important domains for RbpA activity. We have also previously shown that *M. smegmatis* expressing RbpA_*Mtb*_^R88A^ or RbpA_*Mtb*_^R79A^ as its only *rbpA* allele also exhibits a slow growth phenotype due to the importance of RbpA’s interaction with the RNAP and DNA (5). Using the gene swapping approach, we found that the RbpA_*Mtb*_^R10A^, RbpA_*Mtb*_^E17A^, and RbpA_*Mtb*_^R10A/E17A^ point mutants could support viability in *M. smegmatis* and had no effect on growth rate compared to RbpA_*Mtb*_^WT^ in LB media (Fig. 2*A*), indicating that these mutations do not affect RbpA’s essential role in *M. smegmatis*.

To examine the Fdx sensitivity of each *M. smegmatis* strain, we used a zone of inhibition assay, similar to previous studies (2, 11). By spreading approximately 2.5 × 10^8^ colony forming units of bacteria on an agar plate and spotting 10 μl of 100, 250, or 500 μM Fdx dissolved in dimethyl sulfoxide (DMSO) onto a disk placed onto the plate, the bacteria form a lawn after incubation at 37 ^°^C for 2 days, and a zone absent of bacterial growth indicates growth inhibition by Fdx. DMSO had no effect on *M. smegmatis* growth in this assay and did not generate a zone of clearing on its own, whereas incubation of *M. smegmatis* with Fdx resulted in growth inhibition (Fig. 2*B*). We compared the radii of the zones of inhibition formed on each *M. smegmatis* mutant with Fdx and reproduced previous findings that deletion of the RbpA NTT results in resistance to Fdx *in vivo* (RbpA_*Mtb*_^72–111^, RbpA_*Msm*_^28–114^, and RbpA_*Msm*_^72–114^ mutants in Fig. 2, *B* and *C*) (11), which is consistent with the *in vitro* findings (Fig. 1, *B* and *C*). In contrast, the RbpA_*Mtb*_^R10A^, RbpA_*Mtb*_^E17A^, and RbpA_*Mtb*_^R10A/E17A^ mutants were not more resistant to Fdx *in vivo*, despite the trend observed *in vitro* of RbpA_*Mtb*_^R10A/E17A^ displaying decreased Fdx sensitivity compared to RbpA_*Mtb*_^WT^ (Figs. 1, *B* and *C* and 2, *B* and *C*). Strikingly, the *M. smegmatis* RbpA_*Mtb*_^R79A^ and RbpA_*Mtb*_^R88A^ mutants, which have decreased affinity for DNA and the σ factor, respectively, were significantly more sensitive to Fdx treatment (Fig. 2, *B* and *C*). These *in vivo* data highlight the existence of other contributors to RbpA’s effect on Fdx activity that exist in the bacteria but are not recapitulated in the *in vitro* assay.

### Effects on RP_o_ stability correlate to sensitivity to Fdx in M. smegmatis in vivo

**Figure 3:**
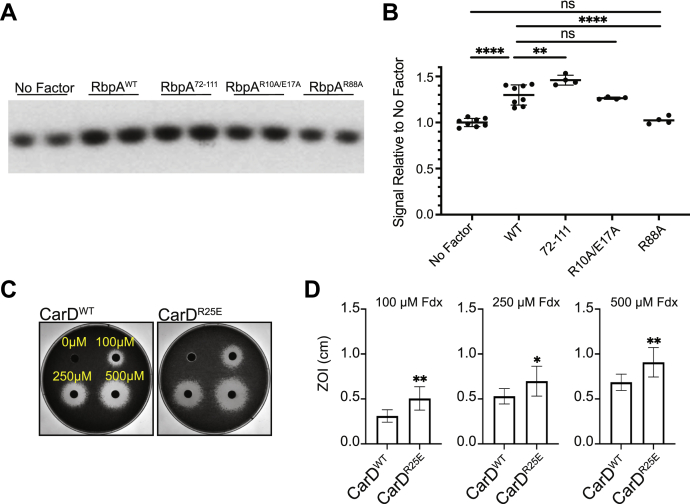
**RP**_**o**_**stability in associated with Fdx sensitivity *in vivo*.***A*, representative gels of three nucleotide transcripts produced by *M. tuberculosis* RNAP-σ^A^ alone or in complex with RbpA_*Mtb*_^WT^, RbpA_*Mtb*_^72–111^, RbpA_*Mtb*_^R10/E17A^, or RbpA_*Mtb*_^R88A^ from a plasmid DNA template containing positions −39 to +4 of *M. tuberculosis rrn*AP3 relative to the +1 transcription start site. *B*, ratio of transcript produced as compared to the average of “No Factor” replicates included on the same gel. Results are plotted as individual values with the mean ± SD shown. Statistical significance of differences was determined by ANOVA and Tukey’s multiple comparison test. ‘ns’, not significant; ∗∗*p* < 0.01; ∗∗∗∗*p* < 0.0001. *C*, zones of inhibition (ZOI) by Fdx on bacterial lawns of *M. smegmatis* expressing CarD_*Mtb*_^WT^ or CarD_*Mtb*_^R25E^ as the only copy of *carD*. *D*, mean radii of ZOI ± SD from at least two experiments with at least three replicates at 100 μM, 250 μM, and 500 μM Fdx is plotted. Statistical significance was analyzed by two-tailed Welch’s *t* test. ∗*p* < 0.05; ∗∗*p* < 0.01. Fdx, fidaxomicin; RbpA, RNA polymerase binding protein A; RP_o_, RNAP-promoter open complex.

Although the RbpA SID and BL domains are not predicted to contact Fdx in structural models, mutations of residues within the SID (R88A) and BL (R79A) still affected Fdx sensitivity *in vivo* (Fig. 2, *B* and *C*). This suggests that the relationship between RbpA and Fdx sensitivity is not limited to the contribution of specific amino acids within the NTT for Fdx binding to RbpA-bound RNAP-σ^A^. Therefore, we investigated whether RbpA’s functional role during transcription initiation contributed to its effects on Fdx sensitivity. During transcription initiation, RbpA stabilizes RNAP-σ^A^ (or σ^B^) RP_o_ (5, 8, 9, 10, 13, 14), which requires binding of the SID to the σ factor and binding of the BL to the DNA (5). In contrast, the NTT and CD antagonize RbpA’s RP_o_ stabilizing activity (5, 10). Using the 3-nucleotide transcription assay to measure RP_o_ stability in the absence of Fdx, we found that addition of RbpA_*Mtb*_^WT^ to *M. tuberculosis* RNAP-σ^A^ and the *rrnA*P3 promoter increased RP_o_ stability compared to no factor, and this effect was abolished with the RbpA_*Mtb*_^R88A^ mutant (Fig. 3, *A* and *B*), consistent with previously published stopped flow fluorescence data (5). Addition of RbpA_*Mtb*_^72–111^ to *M. tuberculosis* RNAP-σ^A^ and the *rrnA*P3 promoter increased RP_o_ stability compared to RbpA_*Mtb*_^WT^, while addition of the RbpA_*Mtb*_^R10A/E17A^ mutant showed similar activity as compared to RbpA_*Mtb*_^WT^ (Fig. 3, *A* and *B*), demonstrating that R10 and E17 are not involved in RbpA’s activity on RP_o_ stability.

The effect of these RbpA alleles on RP_o_ stability mirrors the pattern of Fdx sensitivity *in vivo*, where *M. smegmatis* strains expressing RbpA alleles conferring higher RP_o_ stability (RbpA_*Mtb*_^72–111^) *in vitro* were less sensitive to killing by Fdx. Conversely, *M. smegmatis* strains expressing RbpA alleles conferring decreased RP_o_ stability (RbpA_*Mtb*_^R88A^) *in vitro* were more sensitive to killing by Fdx. This correlation led us to further investigate the relationship between RP_o_ stability and Fdx sensitivity. CarD is another essential transcription factor in mycobacteria that functions to stabilize RP_o_ (1, 7, 8, 10, 35, 36). We reasoned that if RP_o_ stability was linked to Fdx activity *in vivo*, then *M. smegmatis* strains expressing the CarD_*Mtb*_^R25E^ mutant allele, which has a weaker affinity for the RNAP and is defective in stabilizing RP_o_ (2, 7, 35) would be more sensitive to Fdx than *M. smegmatis* expressing CarD_*Mtb*_^WT^. Indeed, when we performed the zone of inhibition assays on these strains, we found that the R25E mutation in CarD also increased the sensitivity of *M. smegmatis* to Fdx (Fig. 3, *C* and *D*). In summary, our experiments uncover a relationship between RP_o_ stability and Fdx sensitivity in *M. smegmatis* (Table 1), suggesting that the role of RbpA for Fdx sensitivity in mycobacteria may involve RbpA’s functional activity during transcription initiation in addition to the role of the RbpA NTT in Fdx binding. In addition, these studies highlight that other factors that regulate RP_o_ stability, such as CarD, could also affect sensitivity to Fdx.

**Table 1:** Summary of the effects of RbpA and CarD mutants on fidaxomicin (Fdx) sensitivity and open complex (RP_o_) stability, compared to WT protein

RbpA construct	*In vitro* Fdx sensitivity	*In vivo* Fdx sensitivity	RP_o_ stability
RbpA^72–111^	Decrease	Decrease	Increase
RbpA^R88A^	No change	Increase	Decrease
RbpA^R10A/E17A^	Decrease^a^	No change	No change
CarD^R25E^	N/A	Increase	Decrease

a The level of decrease in Fdx sensitivity *in vitro* with RbpA^R10A/E17A^ is intermediate to that of RbpA^72–111^, when both are compared to RbpA^WT^.

## Discussion

Prior studies on RbpA have focused almost exclusively on the SID interaction with σ factor and the BL interaction with DNA, leaving the NTT and CD largely uncharacterized. Structural studies have provided tremendous insight into the potential interactions between the NTT and CD with multiple RNAP-σ^A^ holoenzyme domains as well as the antibiotic Fdx (10, 11, 37). Herein, we test the prediction that RbpA R10 and E17 contribute contacts with the antibiotic Fdx that are important for RbpA’s NTT-dependent activity against *M. tuberculosis* RNAP-σ^A^. We find that *in vitro*, combined mutation of both residues affects the IC50 of Fdx activity against the *M. tuberculosis* RNAP-σ^A^ (Fig. 1, *B* and *C*); however, it is still not clear whether RbpA R10 and E17 promote RbpA NTT-dependent Fdx activity through direct interaction with Fdx or through an alternative mechanism. Maintenance of partial Fdx activity against *M. tuberculosis* RNAP-σ^A^ bound by RbpA_*Mtb*_^R10A/E17A^ *in vitro* indicates that additional RbpA NTT residues, or perhaps the entire structural domain, mediate RbpA NTT-dependent Fdx activity. In addition, the RbpA_*Mtb*_^R10A/E17A^ mutant did not alter Fdx sensitivity in *M. smegmatis* (Fig. 2), indicating that those residues play less of a role in Fdx activity *in vivo*. The R88A substitution that weakens RbpA’s interaction with the RNAP *in vivo* (5), and thus would be expected to decrease *M. smegmatis* sensitivity to Fdx since less RbpA would be associated with RNAP-σ^A^, also unexpectedly increased *M. smegmatis* sensitivity to Fdx. Taken together, these observations reveal differences in the effects of RbpA mutants on Fdx sensitivity *in vitro* compared to *in vivo* and support a model where RbpA can impact Fdx activity independent of its direct contacts with the antibiotic.

These discrepancies between the measured sensitivities *in vitro versus in vivo* may be due in part to the limited scope of the *in vitro* assay used here and in previous studies to probe Fdx activity (11), where Fdx is added to RbpA and RNAP-σ^A^ holoenzyme before DNA addition. Whereas in the cell, RNAP-σ^A^ holoenzyme could be bound to DNA prior to Fdx binding. This limitation may bias the *in vitro* assay toward identifying the factors that affect Fdx binding to free RbpA-RNAP-σ^A^ holoenzyme complex. In particular, our *in vivo* results support an association between effects on RP_o_ stability and Fdx sensitivity. Our work indicates that RP_o_ stability is a newly characterized way that RbpA contributes to Fdx activity. During transcription initiation, RP_o_ stabilization involves closing of the RNAP clamp module around downstream nucleic acid as the transcription bubble is formed (38). Structural studies indicate that Fdx inhibits transcription initiation by trapping the mycobacterial transcription initiation complex in an open-clamp conformation (11). In addition, Fdx is predicted to be unable to bind the closed-clamp conformation (11). Therefore, mycobacterial transcription factors such as RbpA and CarD that favor RP_o_ formation (7, 8, 10) may impact Fdx sensitivity by reducing the lifetime of open-clamp RNAP complexes that Fdx can bind. Conversely, Fdx has also been shown to decrease the affinity of CarD to RNAP *in vitro* (39). CarD has a lower affinity to the open-clamp RNAP complex compared to the closed-clamp RNAP complex (RP_o_) (7). Thus, it is possible that Fdx lowers the fraction of CarD bound to RNAP-promoter complexes by reducing the amount of RP_o_ formed at equilibrium. This work highlights the need to biochemically understand Fdx activity against the diversity of RNAP complexes that exist within the bacteria.

In addition to the initiation complexes formed following RNAP-σ^A^ binding to DNA, one could envision other factors that exist *in vivo* and not *in vitro* that could impact Fdx activity. The *in vitro* assays of Fdx activity also exclude RNAP holoenzymes containing alternative σ factors and additional RNAP interacting proteins present in the bacteria. Fdx has been shown to be more active at inhibiting the *E. coli* RNAP-σ^s^ holoenzyme compared to the *E. coli* RNAP-σ^70^ holoenzyme (32), suggesting that the presence of alternative σ factor–bound holoenzymes may also explain some discrepancies between our *in vitro* and *in vivo* findings. In addition to these direct effects on RNAP, truncation of the RbpA NTT and CD results in global dysregulation of gene expression in *M. smegmatis* (5, 10), which could also affect sensitivity to Fdx. Therefore, the effect of RbpA on Fdx activity *in vivo* is likely multifactorial. As such, analysis of RbpA mutants with substitutions in conserved residues within the NTT that are predicted to contact different domains in the RNAP-σ^A^ holoenzyme revealed diverse effects of RbpA on the Fdx sensitivity of *M. smegmatis* (Fig. S1). The impact of these mutants on transcription initiation is unknown, but further investigation into this area could shed more light on how association of RbpA on transcription initiation complexes contributes to antibiotic susceptibility.

Collectively, our results demonstrate that the RbpA NTT domain is a significant contributor to the Fdx sensitivity of the mycobacterial transcription machinery, consistent with previous studies. However, we also discover that the role for RbpA involves more than simply providing amino acids to the Fdx binding site. Our data support a model where multiple RbpA domains, including the NTT, can impact Fdx sensitivity through modulation of transcription initiation kinetics. Our studies reveal a role for another factor that also regulates RP_o_ stability, CarD, in Fdx sensitivity. Fdx is currently used to treat infections caused by *C. difficile*, a bacterium that does not encode an RbpA homolog but does encode CarD and other factors that will regulate transcription by modifying RP_o_ lifetime (1). Therefore, these studies also shed light on pathways that can be targeted to improve Fdx activity in the clinic.

## Experimental procedures

### Media and bacterial strains

All *M. smegmatis* strains were derived from mc^2^155 and grown at 37 ^°^C in LB medium supplemented with 0.5% dextrose, 0.5% glycerol, and 0.05% Tween 80. *M. smegmatis* strains expressing RbpA_*Mtb*_^R4A^, RbpA_*Mtb*_^R4E^, RbpA_*Mtb*_^L6A^, RbpA_*Mtb*_^R7A^, RbpA_*Mtb*_^R7E^ RbpA_*Mtb*_^R10A^, RbpA_*Mtb*_^S15A^, RbpA_*Mtb*_^E17A^ and RbpA_*Mtb*_^R10A/E17A^, RbpA_*Mtb*_^R79A^, RbpA_*Mtb*_^R88A^, RbpA_*Mtb*_^26–111^, RbpA_*Mtb*_^72–111^, RbpA_*Msm*_^28–114^, and RbpA_*Msm*_^72–114^ were engineered using pMSG430 plasmids that express each *rbpA* allele from a constitutive P*myc1-tetO* promoter and integrated into the *attB* site of the *M. smegmatis* Δ*rbpA attB::tet-rbpA* strain previously described (5, 33, 34). The primers used to make RbpA strains are in Table S1. RbpA_*Mtb*_^R79A^, RbpA_*Mtb*_^R88A^, RbpA_*Mtb*_^26–111^, and RbpA_*Mtb*_^72–111^ have been previously described in (5). The *M. smegmatis* Δ*rbpA attB::tet-rbpA* strains expressing RbpA_*Mtb*_^R4A^, RbpA_*Mtb*_^R4E^, RbpA_*Mtb*_^L6A^, RbpA_*Mtb*_^R7A^, RbpA_*Mtb*_^R7E^, RbpA_*Mtb*_^R10A^, RbpA_*Mtb*_^S15A^, RbpA_*Mtb*_^E17A^, RbpA_*Mtb*_^R10A/E17A^, RbpA_*Msm*_^28–114^, RbpA_*Msm*_^72–114^, RbpA_*Mtb*_^R79A^, and RbpA_*Mtb*_^R88A^ were named csm455, csm461, csm456, csm457, csm458, csm451, csm462, csm450, csm498, csm510, csm511, csm322, and csm314, respectively.

### Protein preparation for biochemical assays

Plasmids containing the *M. tuberculosis* H37Rv genomic DNA encoding the different *M. tuberculosis* RNAP holoenzyme subunits were a gift from Jayanta Mukhopadhyay (Bose Institute) (40). Expression and purification were carried out in accordance with the methods described previously (5). Recombinant *M. tuberculosis* RbpA proteins were purified from *E. coli* as previously described using the pET-SUMO vector (primers used to make RbpA constructs for protein purification are in Table S2) (5). RbpA was stored at −80 ^°^C in 150 mM NaCl, 20 mM Tris pH 8.0, and 1 mM β-mercaptoethanol. *M. tuberculosis* RNAP-σ^A^ holoenzyme was stored at −80 ^°^C in 50% glycerol, 10 mM Tris pH 7.9, 200 mM NaCl, 0.1 mM EDTA, 1 mM MgCl_2_, 20 μM ZnCl_2_, and 2 mM DTT.

### Fdx zone of inhibition

*M. smegmatis* cultures were grown to OD_600_ = 0.4 to 0.8. Based on the approximation that OD_600_ = 1.0 is equivalent to 5 × 10^8^ mycobacteria, 2.5 × 10^8^ cells were collected, resuspended in 100 μl of LB, and plated on LB agar plates. Whatman filter paper disks were applied to the plates, and 10 μl of 100 μM, 250 μM, or 500 μM Fdx (Selleck Chemicals) resuspended in DMSO or DMSO alone were added to the Whatman filter paper disks. The plates were incubated at 37 ^°^C for 48 h, and the zones of inhibition were measured. The zone of inhibition for each replicate at each drug concentration is the average of four measurements approximately 90^o^ apart.

### 3-Nucleotide *in vitro* transcription assay

For the Fdx studies in Figure 1, a linear 150 bp dsDNA template containing the *M. tuberculosis rrnA*P3 promoter was prepared by annealing and extending 85-mer oligonucleotide primers (Integrated DNA Technologies) with a 20 nucleotide overlap ranging from nucleotides 1,471,577 to 1,471,726 in the *M. tuberculosis* H37Rv genome (9) and HPLC purified as previously described (7). For the RP_o_ stability assays in Figure 3, a plasmid DNA template containing the *M. tuberculosis rrnA*P3 promoter from the −39 to +4 positions relative to the +1 transcription start site, ranging from nucleotides 1,471,618 to 1,471,660 in the *M. tuberculosis* H37Rv genome, was used. Plasmid DNA was isolated by Midi-prep (Qiagen) and cleaned by alcohol precipitation. For all 3-nucleotide transcription assays, RbpA, *M. tuberculosis* RNAP-σ^A^ holoenzyme, and dsDNA template were incubated at 37 ^°^C for 10 min. Reactions were initiated by adding 2.5 μl of a substrate mixture containing GpU, UTP, and ^32^P radiolabeled UTP and incubating at 37 ^°^C for 10 min to allow for production of a 3-nucleotide product in 20 μl reactions that included a final concentration of 2 μM RbpA (saturating concentration based on (5, 8), 100 nM *M tuberculosis* RNAP-σ^A^ holoenzyme, 10 nM dsDNA template, 1 mM DTT, 0.1 mg/ml BSA (NEB), 200 μM GpU, 20 μM UTP, 0.2 μl of ^32^P radiolabeled UTP, 75 mM NaCl, 10.1 mM MgCl_2_, 2 μM ZnCl_2_, 18 mM Tris pH 8.0, 0.01 mM EDTA, 5% glycerol, and 0.1 mM β-mercaptoethanol. Reactions were stopped with 2X formamide stop buffer (98% [vol/vol] formamide, 5 mM EDTA and 0.05% w/v bromophenol blue). Reaction products were resolved by 22% polyacrylamide-urea gel electrophoresis and exposure to autoradiography film. Products were quantified using ImageJ. Dose–response curves were carried out the same way with the exception that Fdx was added to RbpA and *M. tuberculosis* RNAP-σ^A^ holoenzyme, incubated for 10 min at 37 ^°^C, at which point linear dsDNA template was added and allowed to incubate at 37 ^°^C for 15 min before initiating the reactions with the substrate mixture. The *in vitro* transcription reaction conditions are slightly different than those used in previously published work (11), including different salts in the buffers, different type of holoenzyme preps, and a different dsDNA template, all likely contributing to overall differences in the Fdx IC50 values. Nonetheless, the trends between samples are consistent between this manuscript and previously published work, and therefore, the different reaction conditions do not change the data interpretations or conclusions.

## Data availability

All data are contained in the manuscript and the supporting information file.

## Supporting information

This article contains supporting information.

## Conflict of interest

The authors declare that they have no known competing financial interests or personal relationships that could have appeared to influence the work reported in this paper.

## Supporting information

Supplemental Table S2

Supplemental Figure S1 *A*, alignment of RbpA proteins from actinobacterial species. Conserved RbpA NTT residues targeted in this analysis are indicated by an *asterisk*, and the four RbpA structural domains are indicated. *B*, structural modeling of RbpA NTT interactions with the RNAP from PDB structure 6BZO. RbpA NTT residues targeted in this analysis and the *M. tuberculosis* RNAP-σ^A^ residues positioned to interact with RbpA are shown with PyMol stick representation while the rest of the structure is shown with PyMol cartoon representation. Polar interactions are indicated by *red dashed lines*. *C*, zones of inhibition (ZOI) by Fdx on bacterial lawns of *M. smegmatis* expressing RbpA_*Mtb*_^WT^, RbpA_*Mtb*_^R4A^, RbpA_*Mtb*_^R4E^, RbpA_*Mtb*_^L6A^, RbpA_*Mtb*_^R7A^, RbpA_*Mtb*_^R7E^, or RbpA_*Mtb*_^S15A^ as the only copy of *rbpA*. Mean radii of ZOI ± SD from at least two experiments with at least two replicates at 100 μM, 250 μM, and 500 μM Fdx is plotted. *D*, doubling times of *M. smegmatis* strains expressing RbpA_*Mtb*_^WT^, RbpA_*Mtb*_^R4A^, RbpA_*Mtb*_^R4E^, RbpA_*Mtb*_^L6A^, RbpA_*Mtb*_^R7A^, RbpA_*Mtb*_^R7E^, or RbpA_*Mtb*_^S15A^ normalized to the average doubling time of *M. smegmatis* expressing RbpA_*Mtb*_^WT^. The mean ± SD from at least two independent experiments with at least two replicates per experiment. For both (*C*) and (*D*), statistical significance of differences was analyzed by ANOVA and Tukey’s multiple comparison test. ∗∗*p* < 0.01; ∗∗∗*p* < 0.001; ∗∗∗∗*p* < 0.0001. All comparisons to RbpA_*Mtb*_^WT^ were included in the analysis, but only statistically significant comparisons are indicated in the figure. RbpA_*Mtb*_^WT^ growth rate and ZOI data included in this figure are the same data included in Figure 2, *A* and *C*. Fdx, fidaxomicin; NTT, N-terminal tail; RbpA, RNA polymerase binding protein A; RNAP, RNA polymerase.
