# Supplementary material for: Molecular dissection of RbpA-mediated regulation of fidaxomicin sensitivity in mycobacteria
Source: J Biol Chem. 2022 Feb 19;298(4):101752. doi: 10.1016/j.jbc.2022.101752 (PMC8956947; doi:10.1016/j.jbc.2022.101752)
Supplement: Supplemental Table S1 [file mmc1.docx]

**Supporting Information Table 1** - The primers used to clone RbpA strains into pMSG430 for *M. smegmatis* strain engineering.

| Primer | 5’ to 3’ sequence |
| --- | --- |
| *Mtb rbpA* WT forward primer - XbaI | GTCTAGAATGGCTGATCGTGTCCTGAGGG |
| *Mtb rbpA* WT reverse primer - EcoRI | GGAATTCGGCATCGAGGGACGCCTTTC |
| *Msm rbpA* WT reverse primer - EcoRI | GGAATTCCGTCAGCTCTCGTCAGCTTCC |
| *Mtb rbpA* R4A forward primer - XbaI | GTCTAGAATGGCTGATGCTGTCCTGAGGGGCAGTCG |
| *Mtb rbpA* R4E forward primer - XbaI | GTCTAGAATGGCTGATGAGGTCCTGAGGGGCAGTCG |
| *Mtb rbpA* L6A forward primer - XbaI | GTCTAGAATGGCTGATCGTGTCGCGAGGGGCAG |
| *Mtb rbpA* R7A forward primer - XbaI | GTCTAGAATGGCTGATCGTGTCCTGGCGGGCAGT |
| *Mtb rbpA* R7E forward primer - XbaI | GTCTAGAATGGCTGATCGTGTCCTGGAGGGCAGT |
| *Mtb rbpA* R10A forward primer - XbaI | GTCTAGAATGGCTGATCGTGTCCTGAGGGGCAGTGCCCTCGG |
| *Mtb rbpA* S15A forward overlap primer | GCCTCGGAGCCGTGGCCTATGAGACC |
| *Mtb rbpA* S15A reverse overlap primer | GGTCTCATAGGCCACGGCTCCGAGGC |
| *Mtb rbpA* E17A forward overlap primer | TGAGCTATGCGACCGACCGCAACC |
| *Mtb rbpA* E17A reverse overlap primer | GGTTGCGGTCGGTCGCATAGCTCA |
| *Mtb rbpA* R79A forward overlap primer | GGTTAAGCCGCCCGCGACGCACTGGGA |
| *Mtb rbpA* R79A reverse overlap primer | CCAGTGCGTCGCGGGCGGCTTAAC |
| *Mtb rbpA* R88A forward overlap primer | CATGCTGCTGGAGGCCCGTTCCATCGAAG |
| *Mtb rbpA* R88A reverse overlap primer | CTTCGATGGAACGGGCCTCCAGCAGCATG |
| *Mtb rbpA* 26-111 forward primer - XbaI | GTCTAGAATGGCGCCGCGCCAGATC |
| *Mtb rbpA* 72-111 forward primer - XbaI | GTCTAGAATGCCGAAGAAGGTTAAGCCGCCC |
| *Msm rbpA* 28-114 forward primer - XbaI | GTCTAGAATGGCGCCGCGTCAGGTCGCCCGCTA |
| *Msm rbpA* 72-114 forward primer - XbaI | GTCTAGAATGCCCAAGAAGGTCAAGCCGC |
