# Supplementary material for: Molecular dissection of RbpA-mediated regulation of fidaxomicin sensitivity in mycobacteria
Source: J Biol Chem. 2022 Feb 19;298(4):101752. doi: 10.1016/j.jbc.2022.101752 (PMC8956947; doi:10.1016/j.jbc.2022.101752)
Supplement: Supplemental Table S2 [file mmc2.docx]

**Supporting Information Table 2** - The primers used to clone RbpA strains into pET-SUMO for protein purification.

| Primer | 5’ to 3’ sequence |
| --- | --- |
| *Mtb rbpA* WT forward primer - BamHI | GGGATCCATGGCTGATCGTGTCCTGAGGG |
| *Mtb rbpA* WT reverse primer – HindIII | AAGCTTGGCATCGAGGGACGCCTTTC |
| *Mtb rbpA* E17A forward overlap primer | TGAGCTATGCGACCGACCGCAACC |
| *Mtb rbpA* E17A reverse overlap primer | GGTTGCGGTCGGTCGCATAGCTCA |
| *Mtb rbpA* R79A forward overlap primer | GGTTAAGCCGCCCGCGACGCACTGGGA |
| *Mtb rbpA* R79A reverse overlap primer | CCAGTGCGTCGCGGGCGGCTTAAC |
| *Mtb rbpA* R88A forward overlap primer | CATGCTGCTGGAGGCCCGTTCCATCGAAG |
| *Mtb rbpA* R88A reverse overlap primer | CTTCGATGGAACGGGCCTCCAGCAGCATG |
| *Mtb rbpA* 26-111 forward primer - BamHI | GGGATCCATGCCGCGCCAGATCGCGC |
| *Mtb rbpA* 72-111 forward primer - BamHI | GGGATCCCCGAAGAAGGTTAAGCCGCCCC |
