# Supplementary figures and images for: Molecular dissection of RbpA-mediated regulation of fidaxomicin sensitivity in mycobacteria
Source: J Biol Chem. 2022 Feb 19;298(4):101752. doi: 10.1016/j.jbc.2022.101752 (PMC8956947; doi:10.1016/j.jbc.2022.101752)

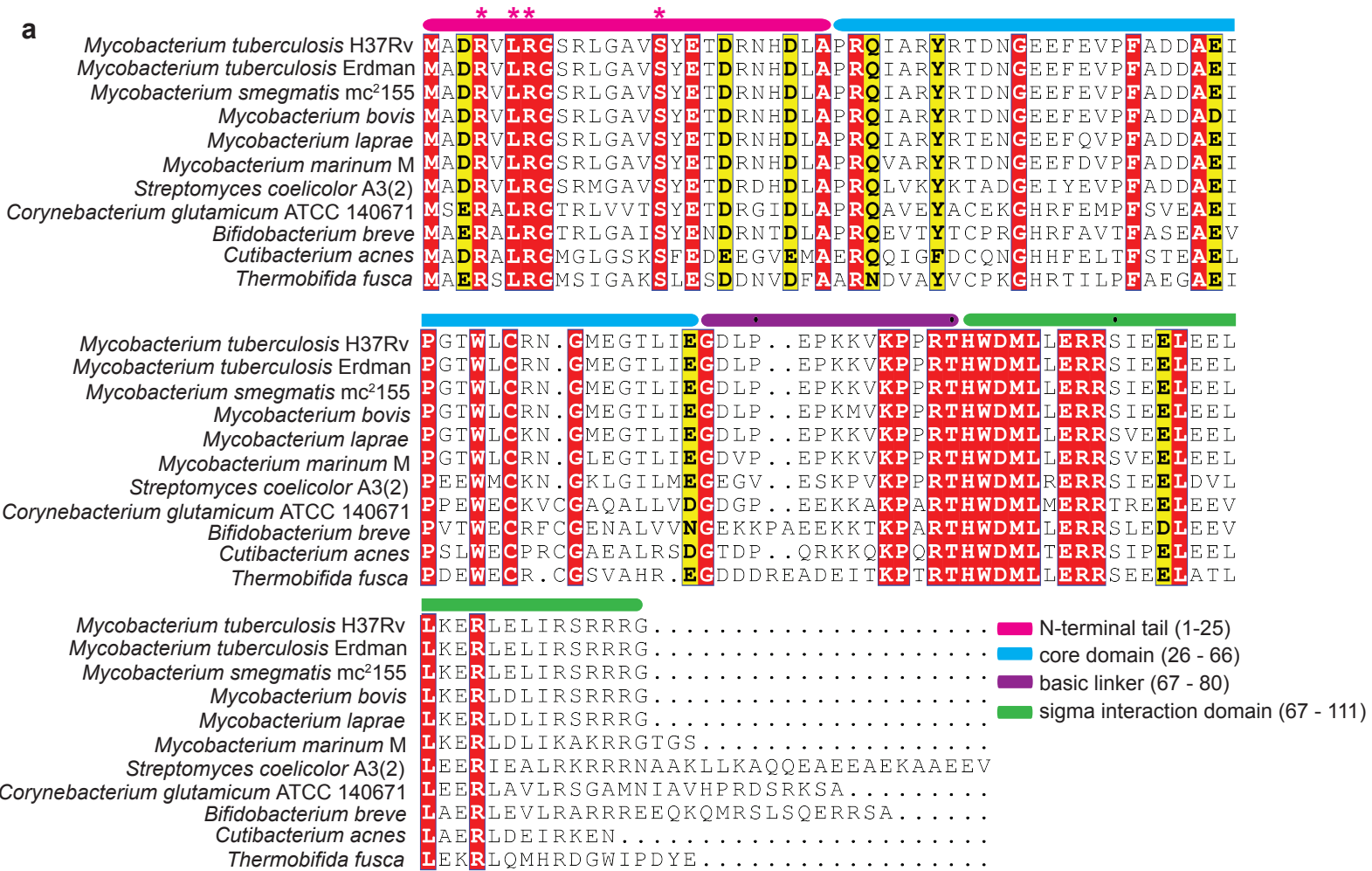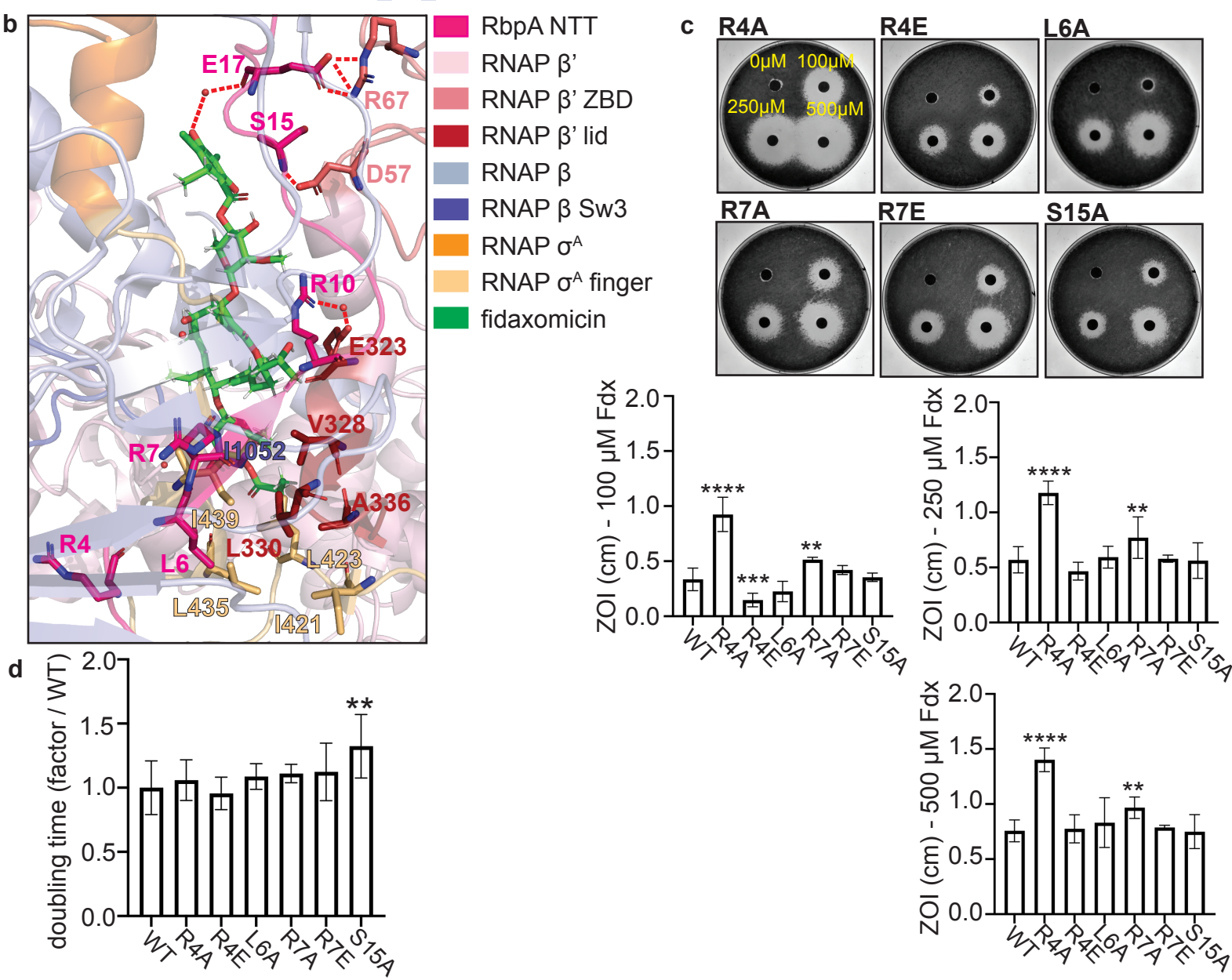

Supplement: Supplemental Figure S1 — A, alignment of RbpA proteins from actinobacterial species. Conserved RbpA NTT residues targeted in this analysis are indicated by an asterisk, and the four RbpA structural domains are indicated. B, structural modeling of RbpA NTT interactions with the RNAP from PDB structure 6BZO. RbpA NTT residues targeted in this analysis and the M. tuberculosis RNAP-σA residues positioned to interact with RbpA are shown with PyMol stick representation while the rest of the structure is shown with PyMol cartoon representation. Polar interactions are indicated by red dashed lines. C, zones of inhibition (ZOI) by Fdx on bacterial lawns of M. smegmatis expressing RbpAMtbWT, RbpAMtbR4A, RbpAMtbR4E, RbpAMtbL6A, RbpAMtbR7A, RbpAMtbR7E, or RbpAMtbS15A as the only copy of rbpA. Mean radii of ZOI ± SD from at least two experiments with at least two replicates at 100 μM, 250 μM, and 500 μM Fdx is plotted. D, doubling times of M. smegmatis strains expressing RbpAMtbWT, RbpAMtbR4A, RbpAMtbR4E, RbpAMtbL6A, RbpAMtbR7A, RbpAMtbR7E, or RbpAMtbS15A normalized to the average doubling time of M. smegmatis expressing RbpAMtbWT. The mean ± SD from at least two independent experiments with at least two replicates per experiment. For both (C) and (D), statistical significance of differences was analyzed by ANOVA and Tukey’s multiple comparison test. ∗∗p < 0.01; ∗∗∗p < 0.001; ∗∗∗∗p < 0.0001. All comparisons to RbpAMtbWT were included in the analysis, but only statistically significant comparisons are indicated in the figure. RbpAMtbWT growth rate and ZOI data included in this figure are the same data included in Figure 2, A and C. Fdx, fidaxomicin; NTT, N-terminal tail; RbpA, RNA polymerase binding protein A; RNAP, RNA polymerase. [file mmc3.pdf]
